# Open versus percutaneous tracheostomy in patients with COVID-19: retrospective cohort analysis

**DOI:** 10.1186/s12890-023-02599-x

**Published:** 2023-08-21

**Authors:** Alejandro González-Muñoz, Camilo Ramírez-Giraldo, Jorge David Peña Suárez, Jaime Lozano-Herrera, Isabella Vargas Mendoza, David Rene Rodriguez Lima

**Affiliations:** 1 Surgery Department, Hospital Universitario Mayor-Méderi, Bogotá, Colombia; 2 Escuela de Medicina Y Ciencias de La Salud, Universidad del Rosario, Bogotá, Colombia; 3 Grupo de Investigación Clínica, Escuela de Medicina Y Ciencias de La Salud, Universidad del Rosario, Bogotá́, Colombia; 4 Critical and Intensive Care Medicine, Hospital Universitario Mayor - Méderi, Bogotá, Colombia

**Keywords:** COVID-19, Tracheostomy, Mechanical ventilation

## Abstract

**Background:**

During the COVID-19 pandemic, a great number of patients required Mechanical Ventilation (MV). Tracheostomy is the preferred procedure when difficult weaning is presented. Surgical techniques available for performing tracheostomy are open and percutaneous, with contradictory reports on the right choice. This paper aims to describe the clinical results after performing a tracheostomy in patients with COVID-19, regarding both surgical techniques.

**Methods:**

An observational, analytical study of a retrospective cohort was designed. All patients admitted to the Hospital Universitario Mayor Méderi, between March 2020 and April 2021 who presented COVID-19 requiring MV and who underwent tracheostomy were reviewed. Open versus percutaneous tracheostomy groups were compared and the primary outcome evaluated was in-hospital mortality.

**Results:**

A total of 113 patients were included in the final analysis. The median age was 66.0 (IQR: 57.2 – 72.0) years old and 77 (68.14%) were male. Open tracheostomy was performed in 64.6% (*n* = 73) of the patients and percutaneous tracheostomy in 35.4% (*n* = 40) with an in-hospital mortality of 65.7% (*n* = 48) and 25% (*n* = 10), respectively (*p* < 0.001). In a multivariate analysis, open tracheostomy technique [OR 9.45 (95% CI 3.20–27.92)], older age [OR 1.05 (95% CI 1.01–1.09)] and APACHE II score [OR 1.10 (95% CI 1.02–1.19)] were identified as independent risk factors for in-hospital mortality. Late tracheostomy (after 14 days) [OR 0.31 (95% CI 0.09–1.02)] and tracheostomy day PaO_2_/FiO_2_ [OR 1.10 (95% CI 1.02–1.19)] were not associated to in-hospital mortality.

**Conclusions:**

Percutaneous tracheostomy was independently associated with lower in-hospital mortality and should be considered the first option to perform this type of surgery in patients with COVID-19 in extended MV or difficulty weaning.

## Introduction

During the COVID-19 pandemic, before massive vaccination, up to 32% of hospitalized patients required Mechanical Ventilation (MV) [1–3], with mortalities of up to 81% in this population [3–5]. MV removal consumes up to 50% of the time of a patient in MV. Tracheostomy is the elective surgery when an extended MV and difficult weaning is anticipated due the use of sedatives and vasoactive agents required in this scenario, and being additionally associated to a lower rate of complications [6–9].

Tracheostomy is recommended in stable patients with extended MV, as it reduces laryngotracheal stenosis, ventilator-associated pneumonia, and hospital stay length [10]. Its advantages include patient comfort, safety, ability to communicate, and improved oral cavity and airway care [11].

Surgical techniques for tracheostomy are open and percutaneous, with contradictory reports about the preferred choice [12–14]. This paper aims to describe the clinical results and complications after tracheostomy in patients with COVID-19, according to both surgical techniques.

## Patients and methods

### Study design

An observational, analytical study in a retrospective cohort was designed. All the electronic records of patients who were admitted to the Hospital Universitario Mayor Méderi, located in Bogotá, Colombia, between March 2020 and April 2021 who required MV, had COVID-19 confirmed by a polymerase chain reaction (PCR) positive result in a nasopharyngeal swab and who underwent tracheostomy were reviewed. This study was reviewed and approved by the Universidad del Rosario's Ethics Committee under number DVO005 2172-CV1656. We followed STROBE guidelines to report this study [15].

### Patients

All patients with PCR-confirmed COVID-19 on a nasopharyngeal swab who were managed with MV and underwent tracheostomy were included in the final analysis. All patients were managed under the institutional protocol based on the evidence available at each of the different moments of the pandemic. Patients under 18 years old and ventilated for a cause other than respiratory failure secondary to COVID-19 were excluded. All included patients received steroids during their hospital stay and protective mechanical ventilation guidelines were followed.

The following variables were analyzed: patients’ demographics; body mass index (BMI); presence of arterial hypertension, diabetes mellitus, chronic obstructive pulmonary disease, chronic kidney disease, cardiovascular disease and malignancy; PaO_2_/FiO_2_ (arterial oxygen partial pressure to fractional inspired oxygen ratio) with which the patient was intubated and with which the tracheostomy was conducted; SOFA and APACHE II scores; number of intubation days before tracheostomy (considering early tracheostomy as the ones performed in under 14 days of MV); type of tracheostomy (open and percutaneous); days of hospital stay and days of intensive care unit (ICU) stay; tracheostomy-associated complications; reintervention and in-hospital mortality; pulmonary mechanics were assessed on the day of mechanical ventilation initiation, considered as day 0 of mechanical ventilation. The first measurement was taken within the first 6 h after orotracheal intubation, and the initial reported measurement occurred on day 4 of mechanical ventilation. These evaluations were conducted in patients receiving volume-controlled ventilation.

### Surgical procedure and decision making

The decision of performing tracheostomy was made jointly by the intensive care physician and the surgeon, in which both clinical course and prognosis was evaluated previous to informed consent signing by the patient’s family. The technique of choice depended on the surgeon's expertise, resources, and the patient’s features such as short neck, obesity, and cervical extension capacity.

Percutaneous tracheostomy was conducted next to the patient's bed in the ICU. In the case of open tracheostomy, this was performed in the operating room. Conscious sedation and neuromuscular relaxation were used in all cases. The staff involved in the procedure used the recommended personal protective equipment. The use of a fiberoptic bronchoscope is not routinely used in our institution during tracheostomy, however all procedures were successful. Early tracheostomy was considered when it was conducted during the first 13 days of MV and late tracheostomy when the time exceeded 14 days.

### Open tracheostomy

A horizontal incision was made 2 fingers length distance above the sternal notch, and the subcutaneous tissue and platysma muscle were incised. Both the sternohyoid and sternothyroid muscles were separated from the midline. Dissection of the pre-tracheal fascia was continued, and the thyroid isthmus was superiorly displaced, in cases where this was not possible the isthmus was divided. Two lateral silk sutures were placed over the second or third tracheal ring, between which the trachea was opened. A tracheostomy tube was then inserted and connected to the ventilator. A thorough review of hemostasis was performed during the procedure [11].

### Percutaneous tracheostomy

Performed using a Tracoe percutaneous tracheostomy device®. A 14-fr needle was inserted with its helix 2-finger lengths above the sternal notch to cannulate the trachea between the second and third tracheal rings. Appropriate placement was verified by aspirating air. The needle was then withdrawn, and the catheter was left in place, through which the guide was passed using the Seldinger technique. The catheter was removed, and a series of dilators were advanced over the guidewire until an appropriate dilatation size was achieved that allowed for passage of the tracheostomy cannula. After placing the tracheostomy cannula, the guidewire was removed, the balloon was inflated, and appropriate positioning was verified using capnography and respiratory noises. Once proper positioning was verified, the orotracheal tube was removed [16].

### Outcomes

The primary outcome evaluated was in-hospital mortality according to the surgical approach used. Secondary outcomes were ICU stay, hospital stay, decannulation (successful tracheostomy removal), and complications according to tracheostomy technique.

### Statistical analysis

A description of demographic, clinical, surgical, and outcome variables was made. The distribution was evaluated using the Shapiro–Wilk and Kolgomorov-Smirnov tests. Categorical variables were described as rates and continuous variables as medians with their corresponding interquartile range (IQR) or means and standard deviations according to normality. Bivariate analyses were conducted related to the primary outcome (in-hospital mortality), technique used (open vs. percutaneous tracheostomy), and tracheostomy time (early versus late). The Chi-squared test was used in the case of categorical variables and the t-test or Mann–Whitney U-test (according to normality) in the case of continuous variables to evaluate differences between the groups, considering a statistically significant difference as a *p* value *r* < 0.05. A multivariate analysis was conducted concerning the outcome of in-hospital mortality, variables with a *p* < 0.25 in the marginal analysis were included in the model. Finally, using automatic selection, a backward stepwise regression model was applied, for which variables with a *p* < 0.1 were considered as independent variables associated to mortality. Potential interactions between the included variables in the final model were tested individually, one by one. Collinearity was evaluated by calculus of the variance inflation factor VIF (Variance Inflation Factor). For variables included in the final model, ORs with their respective 95% confidence intervals were reported. Univariate and bivariate analyses were conducted using SPSS®28, considering a statistically significant *p* < 0.05, the multiple regression model was conducted using RStudio version 4.1.

## Results

**Fig. 1:**
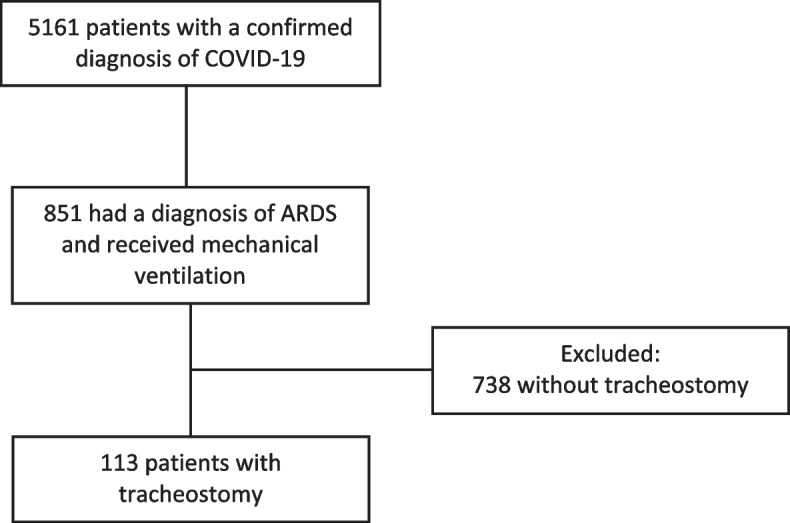
Flowchart of the study selection process

A total of 113 patients were included in the study, the following flowchart (Fig. 1) shows the selection process. 738 patients were not taken for tracheostomy due to non-compliance with prolonged mechanical ventilation criteria, hemodynamic conditions that did not allow for invasive procedures, severe oxygenation disorder and patients who were extubated and deceased during mechanical ventilation. All included patients met ARDS criteria. The distribution was non-normal.

The median age was 66.0 (IQR: 57.2– 72.0) years and 77 patients (68.14%) were male. In-hospital mortality was 51.3% (*n* = 58). Table 1 shows the bivariate analysis concerning the outcome of in-hospital mortality.

**Table 1:** Characteristics of patients with COVID-19 infection who underwent tracheostomy according to in-hospital mortality

	**N (%)**	**Alive *n* = 55 (48.6%)**	**Dead *n* = 58 (51.3%)**	***P* value**
Age (median) (p25-p75)	66.0 (57.5 – 72.00)	61.0 (53.0 – 68.0)	70.0 (61.0 – 74.2)	< 0.001^*^
Sex				0.847
Male	77 (68.1)	37 (67.3)	40 (69.0)	
Female	36 (31.8)	18 (32.7)	18 (31.0)	
BMI (median) (p25-p75)	27.2 (23.5 – 31.2)	27.2 (23.4 – 31.5)	27.2 (23.7 – 31.2)	0.982^*^
Comorbidities				
Arterial hypertension	58 (51.3)	28 (50.9)	30 (51.7)	0.931
Diabetes mellitus	35 (30.9)	19 (34.5)	16 (27.6)	0.424
Cardiovascular disease	12 (10.6)	4 (7.3)	8 (13.8)	0.261
Chronic obstructive pulmonary disease	20 (17.6)	6 (10.9)	14 (24.1)	0.066
Chronic kidney disease	19 (16.8)	6 (10.9)	13 (22.4)	0.102
Oncologic disease	9 (7.9)	3 (5.5)	6 (10.3)	0.337
SOFA (median) (p25-p75)	7.0 (4.5 – 9.0)	7.0 (5.0 – 8.0)	7.0 (3.7 – 9.0)	0.972^*^
APACHE II (median) (p25-p75)	16.0 (12.0 – 21.0)	15.0 (12.0 – 20.0)	18.0 (12.0 – 23.0)	0.097^*^
PaO_2_/FiO_2_ intubation (median) (p25-p75)	79.0 (67.0 – 100.0)	78.0 (67.0 – 98.0)	84.0 (67.0 – 104.7)	0.367^*^
PaO_2_/FiO_2_ tracheostomy (median) (p25-p75)	150 (118.5 – 185.5)	164.0 (137.0 – 194.0)	142.5 (105.5 – 142.5)	0.021^*^
Intubation days (median) (p25-p75)	17.0 (14.0 – 20.5)	17.0 (14.0 – 21.0)	16.5 (13.0 – 20.0)	0.468^*^
Time of tracheostomy				**0.031**
Early	24 (21.2)	7 (12.7)	17 (29.3)	
Late	89 (78.7)	48 (87.3)	41 (70.7)	
Type of tracheostomy				**< 0.001**
Percutaneous	40 (30.0)	30 (54.5)	10 (17.2)	
Open	73 (64.6)	25 (45.5)	48 (82.8)	
Mechanical ventilation Day 0				
Tidal volume (mL) (median) (p25- p75)	420 (400–450)	420 (400–460)	420 (400–440)	0.546^*^
Plateau pressure (cmH_2_O) (median) (p25- p75)	23 (21–25)	23 (21–26)	22 (20–25)	0.485^*^
Compliance static (mL/cmH_2_O) (median) (p25- p75)	32.3 (27.6–44.4)	34.3 (27.0–45.1)	30.7 (28.5–43)	0.673^*^
PEEP (cmH_2_O) (median) (p25- p75)	10 (10–12)	10 (10–12)	10 (10–12)	0.461^*^
Driving pressure (cmH_2_O) (median) (p25- p75)	12 (10–14)	12 (10–14.25)	12 (10–14)	0.824^*^
Mechanical ventilation Day 4				
Tidal volume (mL) (median) (p25- p75)	430 (400–467.5)	425 (400–470)	430 (402–460)	0.915^*^
Plateau pressure (cmH_2_O) (median) (p25- p75)	24 (22–26)	24.5 (21.2–27)	24 (22–24)	0.406^*^
Compliance static (mL/cmH_2_O) (median) (p25- p75)	33.8 (28.2–42)	34.0 (28.1–44.0)	33.8 (31.2–40)	0.857^*^
PEEP (cmH_2_O) (median) (p25- p75)	12 (10–12)	12 (10–12)	10.5 (10–12)	0.403^*^
Driving pressure (cmH_2_O) (median) (p25- p75)	13 (10–14)	13 (10.2–15)	12 (10.5–14)	0.718^*^
Days in hospital (median) (p25-p75)	38.0 (28.0 – 54.0)	50.0 (39.0 – 67.0)	28.0 (22.0 – 36.2)	< 0.001^*^
Days in ICU (median) (p25-p75)	28.0 (22.0 – 38.0)	32.0 (26.0 – 44.0)	25.5 (17.7 – 32.0)	< 0.001^*^
Decannulation	39 (34.5)	37 (67.3)	2 (3.4)	**< 0.001**
Complications				
Bleeding	7 (6.1)	4 (7.3)	3 (5.2)	0.643
Surgical wound infection	2 (1.7)	2 (3.6)	0 (0.0)	0.143
Pneumothorax	1 (0.8)	0 (0.0)	1 (1.7)	0.328
Pneumomediastinum	1 (0.8)	0 (0.0)	1 (1.7)	0.328
Subcutaneous emphysema	2 (1.7)	1 (1.8)	1 (1.7)	0.970
Cardiac arrest	2 (1.7)	0 (0.0)	2 (3.4)	0.165
Laryngotracheal stenosis	1 (0.8)	1 (1.89	0 (0.0)	0.302
Reintervention	6 (5.3)	2 (3.6)	4 (6.9)	0.440

Bold values indicate statistically significant *p*‑values (*p* < 0.05)

*p*‑values were obtained using Fisher’s exact test

^*^p‑values were obtained using the Mann–Whitney test

Older age, lower PaO_2_/FiO_2_ at moment of tracheostomy, moment of tracheostomy and surgical approach were identified as risk factors for mortality in the bivariate analysis (Table 1). There were no significant differences observed in respiratory mechanics between the day of orotracheal intubation (day 0) and day 4 of mechanical ventilation among both the surviving and deceased patients.

Demographical and clinical characteristics and outcomes are shown according to the technique employed (open versus percutaneous) in Table 2. There were no statistically significant differences in pulmonary mechanics on day 0 or day 4 between patients who underwent open tracheostomy and those who underwent percutaneous tracheostomy.

**Table 2:** Characteristics of patients with COVID-19 infection who underwent open versus percutaneous tracheostomy

	**N (%)**	**Percutaneous *n* = 40 (35.3%)**	**Open *n* = 73 (64.6%)**	***P* value**
Age (median) (p25-p75)	66.0 (57.5 – 72.00)	63.0 (56.2 – 71.0)	67.0 (58.0 – 72.5)	0.352^*^
Sex				0.072
Male	77 (68.1)	23 (57.5)	54 (73.9)	
Female	36 (31.8)	17 (42.5)	19 (26.0)	
BMI (median) (p25-p75)	27.2 (23.5 – 31.2)	27.8 (22.5 – 31.7)	27.2 (23.7 – 29.7)	0.810^*^
Comorbidities				
Arterial hypertension	58 (51.3)	20 (50.0)	38 (52.0)	0.834
Diabetes mellitus	35 (30.9)	14 (35.0)	21 (28.7)	0.493
Cardiovascular disease	12 (10.6)	3 (7.5)	9 (12.3)	0.426
Chronic obstructive pulmonary disease	20 (17.6)	7 (17.5)	13 (17.8)	0.967
Chronic kidney disease	19 (16.8)	4 (10.0)	15 (20.5)	0.152
Oncologic disease	9 (7.9)	5 (12.5)	4 (5.4)	0.187
SOFA (median) (p25-p75)	7.0 (4.5 – 9.0)	8.0 (5.2 – 9.0)	6.0 (3.5 – 8.5)	0.017^*^
APACHE II (median) (p25-p75)	16.0 (12.0 – 21.0)	18.0 (13.0 – 23.0)	15.0 (11.5 – 20.5)	0.099^*^
PaO_2_/FiO_2_ intubation (median) (p25-p75)	79.0 (67.0 – 100.0)	83.0 (70.2 – 98.7)	78.0 (65.0 – 100.0)	0.347^*^
PaO_2_/FiO_2_ tracheostomy (median) (p25-p75)	150 (118.5 – 185.5)	162.0 (131.5 – 203.5)	147.0 (113.0 – 180.0)	0.141^*^
Intubation days (median) (p25-p75)	17.0 (14.0 – 20.5)	16.0 (14.0 – 18.0)	17.0 (14.0 – 21.0)	0.233^*^
Time of tracheostomy				0.808
Early	24 (21.2)	9 (22.5)	15 (20.5)	
Late	89 (78.7)	31 (77.5)	58 (79.4)	
Mechanical ventilation Day 0				
Tidal volume (mL) (median) (p25- p75)	420 (400–450)	420 (397.5–457.5)	420 (400–450)	0.746^*^
Plateau pressure (cmH_2_O) (median) (p25- p75)	23 (21–25)	22.5 (21.75–25.25)	23 (20–25)	0.475^*^
Compliance static (mL/cmH_2_O) (median) (p25- p75)	32.3 (27.6–44.4)	31.4 (26.25–39.8)	34.6 (28.5–44.4)	0.367^*^
PEEP (cmH_2_O) (median) (p25- p75)	10 (10–12)	10 (10–12)	10 (10–12)	0.881^*^
Driving pressure (cmH_2_O) (median) (p25- p75)	12 (10–14)	12.5 (10.75–14)	12 (10–14)	0.633^*^
Mechanical ventilation Day 4				
Tidal volume (mL) (median) (p25- p75)	430 (400–467.5)	409 (396.5–470)	430 (412.5–460)	0.261^*^
Plateau pressure (cmH_2_O) (median) (p25- p75)	24 (22–26)	25 (23–27)	23.5 (21.2–25)	0.069^*^
Compliance static (mL/cmH_2_O) (median) (p25- p75)	33.8 (28.2–42)	29.3 (26.6–39.4)	35 (31.4–41.5)	0.137^*^
PEEP (cmH_2_O) (median) (p25- p75)	12 (10–12)	12 (10–12)	12 (10–12)	0.876^*^
Driving pressure (cmH_2_O) (median) (p25- p75)	13 (10–14)	14 (11–15)	12 (10–14)	0.126^*^
Days in hospital (median) (p25-p75)	38.0 (28.0 – 54.0)	44.0 (33.0 – 56.7)	35.0 (24.0 – 50.5)	0.012^*^
Days in ICU (median) (p25-p75)	28.0 (22.0 – 38.0)	29.0 (25.0 – 29.0)	27.0 (20.5 – 35.0)	0.180^*^
Decannulation	39 (34.5)	26 (65.0)	13 (17.8)	**< 0.001**
Complications				
Bleeding	7 (6.1)	1 (2.5)	6 (8.2)	0.228
Surgical wound infection	2 (1.76)	1 (2.5)	1 (1.3)	0.663
Pneumothorax	1 (0.88)	0 (0.0)	1 (1.3)	0.457
Pneumomediastinum	1 (0.8)	1 (2.5)	0 (0.0)	0.175
Subcutaneous emphysema	2 (1.7)	1 (2.5)	1 (1.3)	0.663
Cardiac arrest	2 (1.7)	1 (2.5)	1 (1.3)	0.663
Laryngotracheal stenosis	1 (0.8)	0 (0.0)	1 (1.3)	0.457
Reintervention	6 (5.3)	2 (5.0)	4 (5.4)	0.913
In-hospital mortality	58 (51.3)	10 (25.0)	48 (65.7)	**< 0.001**

Bold values indicate statistically significant *p*‑values (*p* < 0.05)

*p*‑values were obtained using Fisher’s exact test

^*^p‑values were obtained using the Mann–Whitney test

Regarding the employed surgical technique, patients who underwent percutaneous tracheostomy had higher decannulation and lower mortality rates; although they presented a greater hospital stay median time and no differences in ICU stay.

Demographical and clinical characteristics and outcomes are shown according to moment of tracheostomy (open vs. percutaneous) in Table 3.

**Table 3:** Characteristics of patients with COVID-19 infection who underwent early versus late tracheostomy

	**N (%)**	**Early *n* = 24 (%)**	**Late *n* = 89 (%)**	***P* value**
Age (median) (p25-p75)	66.0 (57.5 – 72.00)	68.0 (60.7 – 71.0)	64.0 (56.5 – 73.0)	0.413
Sex				0.861
Male	77 (68.1)	16 (66.6)	61 (68.5)	
Female	36 (31.8)	8 (33.3)	28 (31.4)	
BMI (median) (p25-p75)	27.2 (23.5 – 31.2)	26.2 (23.7 – 29.6)	27.3 (23.4 – 31.5)	0.603^*^
Comorbidities				
Arterial hypertension	58 (51.3)	15 (62.5)	43 (48.3)	0.217
Diabetes mellitus	35 (30.9)	8 (33.3)	27 (30.3)	0.778
Cardiovascular disease	12 (10.6)	1 (4.1)	11 (12.3)	0.248
Chronic obstructive pulmonary disease	20 (17.6)	9 (37.5)	11 (12.3)	**0.004**
Chronic kidney disease	19 (16.8)	5 (20.8)	14 (15.7)	0.553
Oncologic disease	9 (7.9)	1 (4.1)	8 (8.9)	0.439
SOFA (median) (p25-p75)	7.0 (4.5 – 9.0)	6.0 (5.0 – 9.0)	7.0 (4.0 – 9.0)	0.989^*^
APACHE II (median) (p25-p75)	16.0 (12.0 – 21.0)	17.5 (12.0 – 23.7)	15.0 (12.0 – 21.0)	0.471^*^
PaO_2_/FiO_2_ intubation (median) (p25-p75)	79.0 (67.0 – 100.0)	82.5 (68.0 – 115.2)	78.0 (67.0 – 98.5)	0.489^*^
PaO_2_/FiO_2_ tracheostomy (median) (p25-p75)	150 (118.5 – 185.5)	143.5 (99.5 – 179.0)	155.0 (125.0 – 192.0)	0.179^*^
Intubation days (median) (p25-p75)	17.0 (14.0 – 20.5)	12.0 (10.0. – 13.0)	17.0 (15.5 – 22.0)	< 0.001^*^
Type of tracheostomy				0.808
Percutaneous	40 (30.0)	9 (37.5)	31 (34.8)	
Open	73 (64.6)	15 (62.5)	58 (65.1)	
Days in hospital (median) (p25-p75)	38.0 (28.0 – 54.0)	30.5 (19.2 – 45.5)	40.0 (31.0 – 55.00)	0.009^*^
Days in ICU (median) (p25-p75)	28.0 (22.0 – 38.0)	19.5 ( 16.2 – 27.7)	29.0 (25.0 – 39.0)	< 0.001^*^
Decannulation	39 (34.5)	4 (16.6)	35 (39.3)	**0.038**
Complications				
Bleeding	7 (6.1)	2 (8.3)	5 (5.6)	0.624
Surgical wound infection	2 (1.7)	0 (0.0)	2 (2.2)	0.459
Pneumothorax	1 (0.8)	0 (0.0)	1 (1.1)	0.602
Pneumomediastinum	1 (0.8)	0 (0.0)	1 (1.1)	0.602
Subcutaneous emphysema	2 (1.7)	1 (4.1)	1 (1.1)	0.316
Cardiac arrest	2 (1.7)	2 (8.3)	0 (0.0)	**0.006**
Laryngotracheal stenosis	1 (0.8)	0 (0.0)	1 (1.1)	0.602
Reintervention	6 (5.3)	1 (4.1)	5 (5.6)	0.778
In-hospital mortality	58 (51.3)	17 (70.8)	41 (46.0)	**0.031**

Bold values indicate statistically significant *p*‑values (*p* < 0.05)

﻿*p*‑values were obtained using Fisher’s exact test

^*^p‑values were obtained using the Mann–Whitney test

Patients who underwent early tracheostomy had a significantly shorter median hospital stay and ICU stay than patients who underwent tracheostomy after 14 days of MV. However, decannulation rates were lower in patients who underwent early tracheostomy and presented higher mortality rates.

**Fig. 2:**
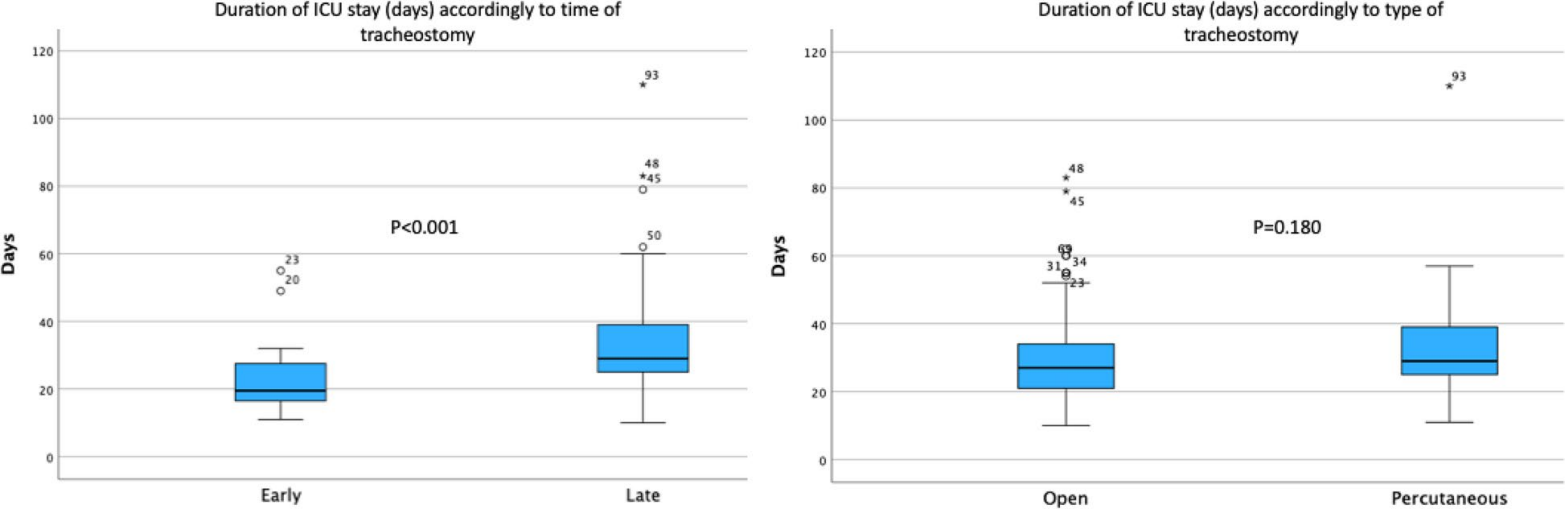
ICU stay (days) according to the moment and tracheostomy approach

Figure 2 shows the length of stay in the ICU according to the moment of the procedure and the surgical approach employed.

**Fig. 3:**
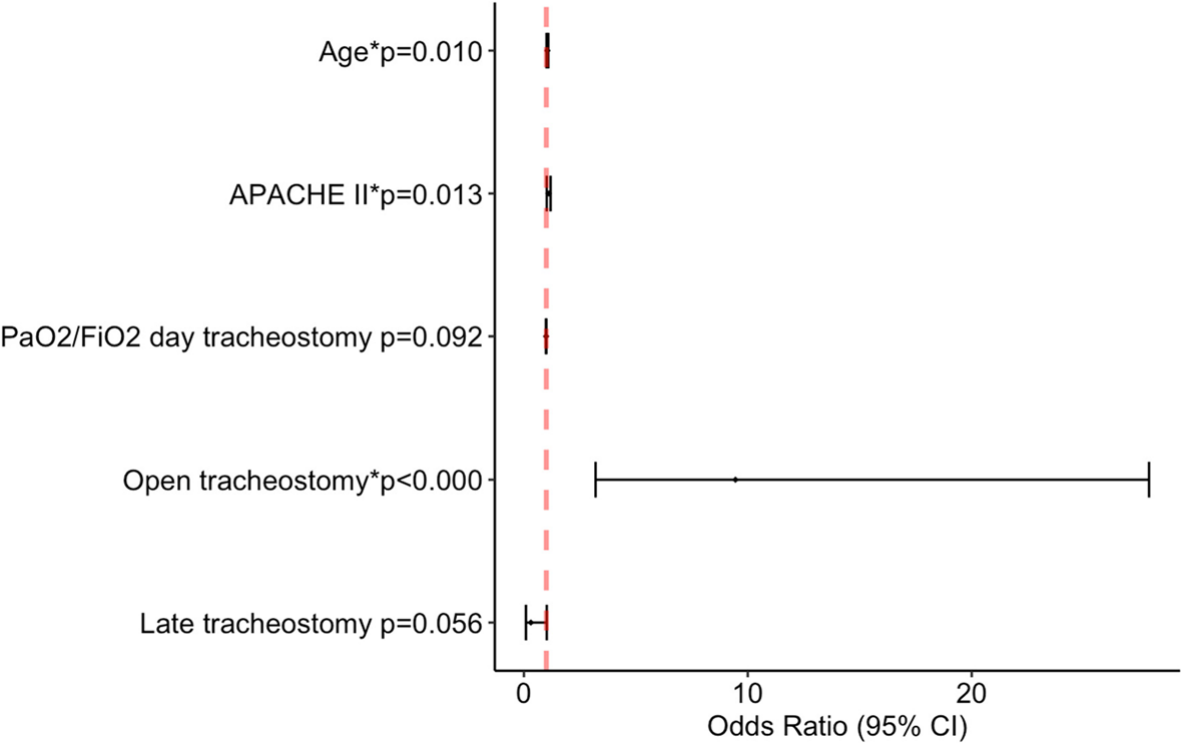
Binomial logistic regression of factors associated with mortality

A multivariate analysis was conducted as described. The results reported that open tracheostomy technique [OR 9.45 (95% CI 3.20–27.92)], age [OR 1.05 (95% CI 1.01–1.09)] and APACHE II score [OR 1.10 (95% CI 1.02–1.19)] were identified as independent risk factors for in-hospital mortality. Late tracheostomy (after 14 days of MV) [OR 0.31 (95% CI 0.09–1.02)] and tracheostomy day PaO_2_/FiO_2_ [OR 1.10 (95% CI 1.02–1.19)] were not associated to in-hospital mortality (Fig. 3). The variance inflation factor VIF of the final model was calculated, and no value was greater than 2, ruling out collinearity between the included variables.

## Discussion

One hundred thirteen patients were included in our study, where we found that overall mortality in patients who underwent tracheostomy was 51.3%. Factors associated with mortality were age, APACHE II score, and surgical approach. Age and APACHE II scores seem logically related to mortality as they evidence higher patient vulnerability, their relation to mortality has also been evidenced in multiple other pathologies.

When comparing the mortality in this study to results reported in different systematic reviews on tracheostomy, ours presented higher rates than average. In a systemic review and the meta-analysis by Ferro, et al., a cumulative mortality of 19.23% (5% CI 15.2% – 23.6%) was reported [17]; in a systemic review and the meta-analysis by Benito, et al., a cumulative mortality of 13.1% (95% CI 8.48%—18.44%) was reported [7], and in a systemic review and the meta-analysis made by Battaglini, et al., a mortality of 22.1% (95% CI 18.7- 25.5) was reported [18]. Only a few studies have reported a mortality between 53.7% y 57.8%, higher than the one found in our study [19–21]. This may be explained by the older age of patients in our cohort and because the majority were attended during the most catastrophic peak of the pandemic in Colombia.

When comparing moment of tracheostomy (early versus late), a statistically significant difference was found in our study in favor of early tracheostomy in terms of shorter hospital stays and shorter ICU stays. In terms of mortality, the bivariate analysis showed a higher mortality for early tracheostomy, however, there were no statistically significant differences in the multivariate analysis. In a systematic review and meta-analysis performed by Ji, et al., a shorter ICU stay was evidenced in early tracheostomies, with no statistically significant differences between early and late tracheostomy regarding mortality [22]. The decision to perform either late or early tracheostomy depended on changing recommendations throughout the pandemic’s course and resources available at the moment.

When evaluating percutaneous versus open tracheostomy, open tracheostomy was found to be a factor associated with mortality; the reason for this difference could be a lower PaO_2_/FiO_2_ ratio with which procedures were conducted. Patients who underwent percutaneous tracheostomy had a higher PaO_2_/FiO_2_ ratio (162.0 IQR: 131.5 – 203.5) than patients who underwent open tracheostomy (147.0 IQR: 113.0 – 180.0), however this variable showed no statistically significant differences in the multivariate analysis. Another factor that could explain this finding is that patients selected for open tracheostomy had unfavorable anatomical structures, such as short neck and obesity considering the latter is also a risk factor for mortality due to Sars-CoV2 infection [23]; however, there were no statistically significant differences in BMI between both techniques. Additionally, it is important to note that some factors associated with severity of Sars-Cov2 infection were not taken into account that could explain a higher mortality in one of the groups [24]. Furthermore, the SOFA score was higher with statistically significant differences in patients taken to open tracheostomy. However, a retrospective observational study involving 72 patients reported a mortality rate of 92% in patients who underwent open tracheostomy compared to 65.9% in the percutaneous tracheostomy group in patients with Sars-Cov2 infection [12], suggesting that the technique employed may influence mortality, as evidenced in the results of this study.

In a pre-pandemic systematic review and meta-analysis by Iftikhar, et al., three different percutaneous approaches and open tracheostomy were compared reporting no statistically significant differences in major complications [13]. Another systematic review and meta-analysis by Klotz, et al. before the pandemic also found no statistically significant differences in mortality between the two surgical approaches [14].

In a systematic review and meta-analysis by Ferro, et al., mortality between both surgical techniques was compared (percutaneous versus open) in patients with Sars-CoV2 infection, reporting no statistically significant differences (RR 1.96 95% CI 0.19 – 20.37); however, this study only included 4 studies for a total of 250 percutaneous tracheostomies and 172 open tracheostomies [17].

This study recognizes some limitations such as using data from a single high complexity center exclusively, which limits the generalization of the results; its retrospective nature which may influence selection bias; being conducted on patients with COVID-19 only, which also does not allow to generalize these findings in patients using MV suffering from other etiologies; in addition, despite the use of steroids in all patients and the use of protective mechanical ventilation, the therapies used on all patients were not specifically analyzed (including the position of the patient).

## Conclusions

Percutaneous tracheostomy was independently associated with lower in-hospital mortality and should be considered as the first option for performing tracheostomy on COVID-19 positive patients in extended MV or with difficult weaning. The non-mobilization of the patient to the operating room, its performance at the patient's bedside, speed, and low rate of complications may explain these findings.

## Data Availability

The data used in the present study are available upon request to the corresponding author.

## Abbreviations

VM: Mechanical ventilation
PCR: Polymerase chain reaction
PaO_2_/FiO_2_: The ratio of arterial oxygen partial pressure to fractional inspired oxygen
VIF: Variance Inflation Factor
ICU: Intensive care unit
